# Wnt1 Inhibits Hydrogen Peroxide-Induced Apoptosis in Mouse Cardiac Stem Cells

**DOI:** 10.1371/journal.pone.0058883

**Published:** 2013-03-22

**Authors:** Jingjin Liu, Yongshun Wang, Wenjuan Du, Wenhua Liu, Fang Liu, Lulu Zhang, Maomao Zhang, Meng Hou, Kai Liu, Shuo Zhang, Bo Yu

**Affiliations:** 1 Cardiology Department, Second Affiliated Hospital of Harbin Medical University, Harbin, Province Heilongjiang, China; 2 Key Laboratories of Education Ministry for Myocardial Ischemia Mechanism and Treatment, Harbin, Province Heilongjiang, China; 3 Intensive Care Unit (ICU) Department, Second Affiliated Hospital of Harbin Medical University, Harbin, Province Heilongjiang, China; University of Cincinnati, United States of America

## Abstract

**Background:**

Because of their regenerative and paracrine abilities, cardiac stem cells (CSCs) are the most appropriate, optimal and promising candidates for the development of cardiac regenerative medicine strategies. However, native and exogenous CSCs in ischemic hearts are exposed to various pro-apoptotic or cytotoxic factors preventing their regenerative and paracrine abilities.

**Methods and Results:**

We examined the effects of H_2_O_2_ on mouse CSCs (mCSCs), and observed that hydrogen peroxide (H_2_O_2_) treatment induces mCSCs apoptosis via the caspase 3 pathway, in a dose-dependent manner. We then examined the effects of Wnt1 over-expression on H_2_O_2_-induced apoptosis in mCSCs and observed that Wnt1 significantly decreased H_2_O_2_-induced apoptosis in mCSCs. On the other hand, inhibition of the canonical Wnt pathway by the secreted frizzled related protein 2 (SFRP2) or knockdown of β-catenin in mCSCs reduced cells resistance to H_2_O_2_-induced apoptosis, suggesting that Wnt1 predominantly prevents H_2_O_2_-induced apoptosis through the canonical Wnt pathway.

**Conclusions:**

Our results provide the first evidences that Wnt1 plays an important role in CSCs’ defenses against H_2_O_2_-induced apoptosis through the canonical Wnt1/GSK3β/β-catenin signaling pathway.

## Introduction

Accumulating evidences during the past decade in both humans and animal models documented the presence of endogenous cardiac stem cells (CSCs) in adult myocardium. [1], [2], [3], [4] In response to local tissue injury, CSCs differentiate into specialized cells, while the pool of stem cells is maintained in time through self-renewal and enhanced proliferation. [5], [6] Exogenous CSCs transplantation into 30-day infarcted rat hearts was shown to activate endogenous CSCs, thus alleviating left ventricular dysfunction. [7] Furthermore, human cardiosphere-derived cells were reported to exhibit paracrine abilities through the secretion of growth factors, resulting in anti-apoptotic effects on surviving cardiomyocytes following their intra-myocardial injection after myocardial infarction MI in mice. [8] Therefore, because of their direct regenerative and paracrine abilities, the use of CSCs is considered highly promising as the most appropriate and optimal candidate cell type for future cardiac regenerative medicine studies and strategies. The c-kit-positive (c-kit^+^) CSCs are the only adult-derived CSCs known to exhibit all the stem cells characteristics, being clonogenic, self-renewing, multipotent and having substantial regenerative potential in an *in vivo* animal MI regeneration assay [1].

Native and exogenous stem cells in ischemic hearts are exposed to various pro-apoptotic and cytotoxic factors. Furthermore, during repopulation and differentiation, a number of newly produced cells may die by apoptosis during neocardiovascular tissue remodeling and morphogenesis. However, the molecular cues and signaling pathways modulating CSCs apoptosis in tissue injury, especially in MI, remain unclear.

Wnt are secreted as glycosylated lipid-modified cysteine-rich proteins and play crucial roles in embryonic development, morphogenesis, cell proliferation, differentiation and migration. [9], [10], [11] Wnt signaling includes the canonical (Wnt/β-catenin and cTnnB1) and non-canonical (Wnt/Ca^2+^) pathways. [12], [13] A less described Wnt pathway is the Wnt-JnK pathway. [14] Several reports proposed time- and context-dependent roles for canonical Wnt signaling in cardiogenesis and progenitor cell biology. Canonical Wnt signaling exhibits biphasic and antagonistic effects on cardiomyogenesis and hematopoiesis/vasculogenesis, depending on the stage of development. [15], [16], [17], [18], [19], [20], [21] Also, some researchers showed biphasic and antagonistic effects on apoptosis in cardiac cells and stem cells. Studies using mesenchymal stem cells (MSC) demonstrated that Wnt3a, a canonical Wnt, reversed acetylsalicylic acid-induced MSC apoptosis. [22] On the other hand, canonical Wnt signaling was also shown to promote apoptosis; however, this pathway is prevented by SFRP2 through direct binding in H9C2 cells. [23] Caspase 3 is the most extensively studied apoptotic protein. Caspase 3 is synthesized as an inactive proenzyme that is processed in cells undergoing apoptosis by self-proteolysis and/or cleavage by another upstream protease.

Since the Wnt/β-catenin signaling pathway is crucial in adult mammals for regulating cell proliferation, cell fate, apoptosis, and axis polarity induction, [24] we aimed to investigate the role of Wnt pathways in the oxidative stress-induced CSCs apoptosis. First, we examined if hydrogen peroxide (H_2_O_2_) treatment induced CSCs apoptosis via the caspase pathway. Secondly, we examined if Wnt1 is involved in the H_2_O_2_-induced CSCs apoptosis. This present study is the first to examine Wnt1 critical role as a cardiac protective agent against H_2_O_2_-induced CSCs apoptosis.

## Materials and Methods

### CSCs Isolation and Culture from Adult Babl/c Mice Hearts

CSCs were isolated from the hearts of Babl/c mice (18–25 g) using a previously published method with a minor modification [1]. All Babl/c mice were obtained from the Laboratory Animal Science Department, the Second Affiliated Hospital of Harbin Medical University, Heilongjiang, PR China. All experimental animal procedures were approved by the Local Ethical Committee of Harbin Medical University Animal Care and Use. Briefly, mice were injected with heparin (5,000 IU/kg, i.p.) 20 min prior to the experimental protocol, and were then killed by cervical dislocation. The heart was excised and the aorta was rapidly cannulated. The cannulated heart was mounted on a Langendorff perfusion apparatus with constant flow and perfusion pressure was monitored. The heart was firstly perfused with Ca^2+^-free Tyrode solution for 10 min to remove the blood, and was then digested using 0.5 mg/ml collagenase (Sigma, St-Louis, MO, USA) and 0.5 mg/ml trypsin (GIBCO, Invitrogen Inc., Carlsbad, CA, USA) at 37°C for 30 min. The heart tissue was then chopped and the cell suspension was filtered with a Steriflip (SCNY00100-1EA, Millipore corp., Billerica, MA, USA). Cells were incubated with a FITC rat anti-mouse CD117/c-kit antibody (BD Biosciences, Franklin Lake, NJ, USA) and separated using MACS anti-FITC microbeads (Miltenyi Biotec, Bergisch Gladbach, Germany). Small round cells, containing most of the c-kit^+^ population, were obtained and were cultured for 3–5 days in HyClone Dulbecco’s MEM/F12 (Thermo Fisher Scientific, Waltham, MA, USA) containing fetal bovine serum (FBS), 10 ng/ml bFGF (PeproTech, Rocky Hill, NJ, USA), 10 ng/ml IGF (PeproTech) 10 ng/ml, EGF (PeproTech) and 10 ng/ml LIF (Sigma), at 37°C. After recovery, cells were used for subsequent experiments.

### Construction of pEGFP-C3-Wnt1 Fusion Expression Plasmid and Transfection into Mouse CSCs

Wnt1 full-length cDNA was cloned by RT-PCR from total RNA extracted from mouse CSCs (mCSCs) using the following primer pair: 5′-GCAAGCTTATGAGGTGGCTCCTGCCC-3′ (forward) and 5′-GCGGTACCCTAATTGGCAATCTCTTCGAAGTC-3′ (reverse). cDNA was purified and ligated to pGEM-T-easy vector (Promega, Madison, WI, USA). The insert was digested using the SacII enzyme and ligated into pEGFP-C3 to generate the fusion expression vector, pEGFP-C3-Wnt1. Orientation and integrity of the inserted Wnt1 cDNA sequence and the continuation of the open reading frame of the pEGFP-C3-Wnt1 fusion peptide were confirmed by restriction enzyme digestion (EcoRI) and by sequencing. mCSCs were plated on day 1 at 20,000 cells/well in a 6-well plate (∼15% confluency). On day 2, culture medium (no antibiotics) was changed and culture was continued for 2–4 h. Plasmid transfection was performed by mixing 100 µl HyClone DMEM medium (Thermo Fisher Scientific) with 6 µl HP DNA Transfection Reagent (Roche Applied Science, Penzberg, Germany) for 5 min, then by adding 2 µg DNA to the HP-DMEM mix, incubating for 15 min and transferring with 1000 µl complete medium (no antibiotics). On day 3, fresh culture medium was provided. On days 3–5, cells were tested by real-time PCR and flow cytometry analysis to confirm the expression of pEGFP-C3-Wnt1.

### Small Interfering RNA (siRNA) Knockdown of β-catenin

Gene silencing by small interfering RNA (siRNA) uses a small double-strand RNA that degrades target mRNA. β-catenin siRNA duplex were synthesized by Shanghai GenePharma Co., Ltd (Shanghai, China) (sense: 5′-GCCUCUGAUAAAGGCAACUTT-3′; antisense: 5′-AGUUGCCUUUAUCAGAGGCTT-3′). In the control group, cells were treated with transfection reagents (vehicle) or with a non-targeting siRNA (siRNA-NC) (sense: 5′-UUCUCCGAACGUGUCACG-3′; antisense: 5′-ACGUGACACGUUCGGAGAATT -3′).

Cells were transfected using the X-treme siRNA Transfection Reagent (Roche Applied Science, Penzberg, Germany), according to the manufacturer’s instructions. In brief, mCSCs were plated in a 6-well plate and treated with the X-treme GENE siRNA Transfection Reagent in a 5∶1 ratio to the siRNA mass for 20 min. Cells were then transfected with a mixture containing 150 µM siRNA and incubated in 2 ml of FBS-free Opti-MEM medium (Invitrogen Inc., Carlsbad, CA, USA) for 4–6 h. Transfection efficiency was analyzed by western blot analysis, and revealed an >80% reduction of β-catenin. Transfection efficiency was also analyzed by fluorescence analysis using a confocal laser-scanning microscope (Fluo View v5.0 FV300; Olympus Corporation, Tokyo, Japan) and counted in 10 randomly selected fields. After a 48 h growth in conditioned medium, transfected cells were subjected to analysis.

### Treatment of the Mouse CSCs with H_2_O_2_

After transfection with pEGFP-C3-Wnt1 fusion plasmid and negative control (pEGFP-C3) for 72 h, medium was replaced with 1000 µl of fresh DMEM medium without serum and culture was continued at 37°C for 1 h. Different concentrations of H_2_O_2_ were prepared in DMEM medium without serum. Final H_2_O_2_ concentrations were 0, 50, 100, 150, 200, 250, 300 µM. medium was replaced with this new medium and cells were cultured for 2 h.

### Immunofluorescence

To characterize the c-kit^+^ cells among the isolated cells, cells were fixed with 4% (v/v) formaldehyde for 15 min. After washing with PBS, cells were blocked with 10% BSA, and incubated at 37°C for 1 h with rabbit anti-c-kit (1∶100, BA0467, Boster Bioengineering Co., Wuhan, China), rabbit anti-GATA4 (1∶100, bs-1778R, Boster Bioengineering Co.) and mouse anti-desmin antibodies (1∶100, BM0036, Boster Bioengineering Co.). Mouse anti-cardiac myosin heavy chain antibodies (1∶100, ab15, Abcam Ltd., Cambridge, MA, USA) and mouse anti-Tn-I (1∶100, ab19615, Abcam Ltd.). Cells were then washed and incubated in the dark for 1 h at 37°C with Rhodamine (TRITC)-conjugated AffiniPure Goat Anti-Mouse IgG (H+L)- antibodies (1∶200, ZSGB-BIO, Beijing, China) and Fluorescein (FITC)-conjugated AffiniPure Goat Anti-Rabbit IgG (H+L) antibodies (1∶200, ZSGB-BIO, Beijing, China). After washing, nuclei were counterstained with 4′,6-diamidino-2-phhenylindole dihydrochloride (DAPI, Sigma, St-Louis, MO, USA). Cells were examined under a fluorescence microscope (DMI4000B, Leica, Germany).

### Quantitative RT-PCR

Total RNA was extracted from mCSCs after various combinations with or without pEGFP-C3-Wnt1 fusion plasmid and SFRP2 treatment. After pretreatment using RNase-free DNase I, 2 µg of total RNA was used for cDNA synthesis using a ThermoScript RT-PCR System (Invitrogen). Real-time RT-PCR experiments were performed on a Capillary Lightcycler (Roche Applied Science) using a Roche Fast Start SYBR Green I Kit. Genes amplification was confirmed by calculating melting temperatures (Tm) for the products from the melting peak curve (−dF/dT vs. temperature). All amplicons were collected and confirmed by agarose gel electrophoresis and sequencing. A standard curve of cross-point versus logarithmic concentration was created using one of the cDNA samples with serial dilutions or with known concentrations of plasmid DNA with a Wnt1 gene insert. Negative controls were included, using cDNAs synthesized in the same way as above but with no reverse transcriptase. Each cDNA sample was run in triplicate. Data were averaged and standard deviations were calculated. The GAPDH gene was used as the standard control. The primers used in this study and PCR conditions are described in Table 1.

**Table 1:** PCR primers and PCR conditions.

Primer	Annealing temperature °C
Wnt1	GAPDH
5′-GCAAGCTTATGAGGTGGCTCCTGCCC-3′;	5′-GGCACAGTCAAGGCTGAGAATG-3′;
5′-GCGGTACCCTAATTGGCAATCTCTTCGAAGTC-3′′;	5′-ATG GTG GTG AAG ACG CCA GTA-3′;
55	55

### Western Blotting

Protein samples were denatured at 95°C for 5 min before loading onto an SDS–polyacrylamide gel (reducing or non-reducing, 4–15%) and run under standard conditions. Proteins were transferred onto polyvinylidene difluoride (PVDF, Millipore) membranes. Membranes were blocked with 5% non-fat milk at room temperature for 1 h in Tris-buffered saline containing Tween 20 (TBST). Primary antibodies against GSK-3β, p-GSK-3β, caspase 3 and β-catenin (Cell Signaling, Danvers, MA, USA), or β-actin (Zhongshan Goldenbridge Biotechnology Co. Ltd., Beijing, China) were incubated overnight with the membranes at 4°C. Membranes were then incubated 60 min with peroxidase-conjugated Affinipure goat anti-rabbit IgG (H+L) and anti-mouse IgG (H+L)-labeled secondary antibodies diluted 1∶2000. Membranes were washed in TBST solution with 0.5% Tween-20 before the ECL detection with BeyoECL Plus (Beyotime Institute of Biotechnology, Haimen, China). After exposure on an X-ray film, the blot was stripped in 5 ml sripping buffer (CoWin Biotech, Beijing, China) for 15 min at room temperature and hybridized using the antibody against β-actin for normalization. The protein band densitometry was analyzed using the Tanon Gel Imaging System (Shanghai Tanon Co. Ltd., Shanghai, China).

### TUNEL Assay

Terminal deoxynucleotidyl transferase-mediated dUTP nick-end labeling (TUNEL) staining was performed using an *in situ* cell death detection kit (Roche) according to the manufacturer’s protocol. The c-kit^+^ cells were fixed in 4% paraformaldehyde and stained. TUNEL-positive cells were imaged using a confocal laser-scanning microscope (Fluo View v5.0 FV300; Olympus Corporation, Japan) and counted in 10 randomly selected fields. Results are expressed as the proportion of TUNEL-positive cells to total cells counted. DAPI (1 µg/mL) was used for nuclear counterstaining.

### Flow Cytometry

#### Surface marker characterization

A single cell suspension of 0.5–1.0×10^6^ cells/ml in phosphate buffered saline (Ca^+2^/Mg^+2^free), were incubated in 1∶100 dilution at 4°C for 30 minutes in the dark for tagging with fluorescent primary antibodies: CD29-FITC, CD90-PE, c-kit(CD117)-FITC, sca-1-FITC, and CD34-PE (BD Biosciences, Franklin Lake, NJ, USA). A total of 10,000 events were acquired using a BD LSRII flow cytometer (BD Biosciences) and data were analyzed using the BD FACSDiva™ software. Flow cytometry was carried out using cells from three independent experiments and was performed in duplicates.

#### Transfection efficiency assessment

After plasmid transfection, cells were cultured and harvested after 72 h. The cellular uptake of plasmid was determined by flow cytometry.

#### Apoptosis detection

CSCs apoptosis was assessed using the annexin V-FITC apoptosis detection kit, according to manufacturer’s instructions (BD Biosciences). Briefly, CSCs were grown in the presence or absence of H_2_O_2_ for 2 hours after transfection with pEGFP-C3-Wnt1 plasmid. CSCs were cultured in DMEM/F12 with 10% FBS as negative controls. Cells were harvested and washed once in PBS and resuspended in buffer and incubated with annexin V-FITC in the dark at room temperature for 30 minutes. Cells were then washed once with PBS and resuspended in buffer supplemented with propidium iodide (PI).

### Statistical Analysis

Statistical analysis was performed with SPSS 18.0 (SPSS Inc., Chicago, IL, USA). Measurements are presented as mean ± SD. Comparisons for all pairs were performed by the Student’s t-test or the least significant difference (LSD) test. A p-value <0.05 was considered significant.

## Results

### Characterization of Mouse c-kit^+^ Cardiac Cells

**Figure 1:**
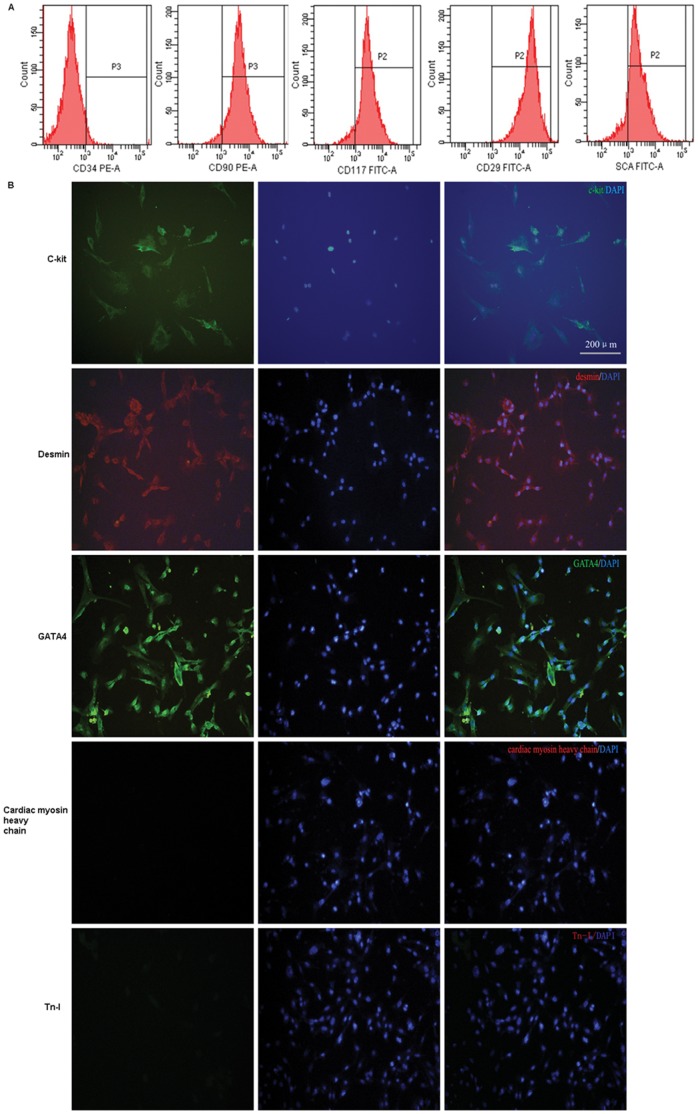
Flow cytometry and Immunofluorescence analysis of c-kit+ cells. (A) Flow cytometry analysis of c-kit+ cells. The c-kit+ cells were enriched by the MACS system with FITC-conjugated anti-c-kit antibody and anti-FITC microbeads; after sorting, 99% of the cells expressed c-kit. The c-kit+ cells were stained with PE-conjugated anti-CD90 and anti-CD34 antibodies, and with FITC-conjugated anti-Sca-1 and anti-CD29 antibodies. In enriched c-kit+ cells, ∼99% of the cells expressed CD90, ∼95% of the cells expressed CD29, ∼3% of the cells expressed CD34, and ∼87% of the cells expressed Sca-1. (B) Immunofluorescence analysis of c-kit+ cells. Fresh mCSCs in culture were fixed and stained for indirect immunofluorescence. Nuclei were counterstained with DAPI. Most of the cells were c-kit-positive. The c-kit+ cells express desmin- (27%±9%) and GATA-4-positive (49%±11%), and no staining for cardiac myosin heavy chain or cardiac TnI was detected.

Cardiac cells were isolated from Babl/c mice (18–25 g). Small cells, containing most of the c-kit+ population, were separated from the differentiated myocytes by differential centrifugation and incubated with a FITC rat anti-mouse CD117/c-kit antibody. The c-kit+ cells were separated either by repeated passing, using MACS. After purification, flow cytometry showed that 99.4±0.4% of cells were c-kit+ (Figure 1A), but were negative for CD34 (1.1±0.42%). In the meanwhile, as expected, these cells were positive for the control mesenchymal marker CD90 (95.4±0.7%), CD29 (99.4±0.5%) and the stemness marker sca-1 (87.4±2.07%). These results suggested that mCSCs, although maintaining stemness phenotypic features, displayed some characteristics of the early cardiac phenotype (Figure 1A).

Immunofluorescence microscopy of c-kit-enriched mCSCs was performed and confirmed previous data, showing that a large number of cells expressed c-kit, GATA-4 and desmin. No markers characterizing fully differentiated cardiomyocytes, such as cardiac myosin heavy chain and cardiac troponin I (cTnI), were detected (Figure 1B).

### Establishment of Wnt1 Gene Overexpression in mCSCs

**Figure 2:**
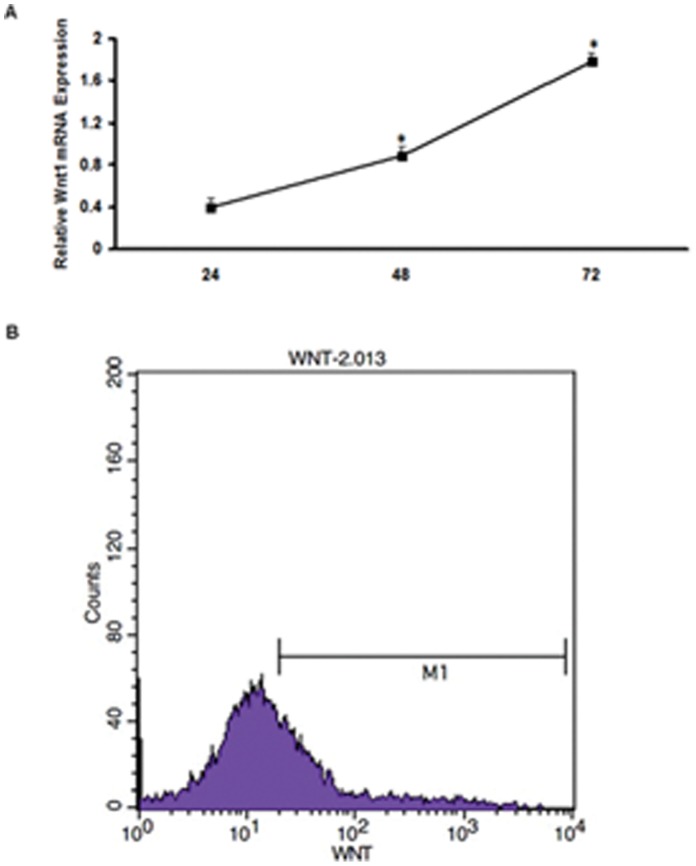
The pEGFP-C3-Wnt1 fusion plasmids were transfected in mCSCs. (A) Total RNA was isolated from Wnt1-CSCs. Real-time PCR confirmed that the optimal time for Wnt1 expression after plasmid transfection was 72 h. (B) Confirmation of plasmid transfection efficiency by the presence of green fluorescence, performed by flow cytometry analysis. Green fluorescence intensities were measured in transfected cells by excitation at 488 nm and emission recorded at 530 nm. Each value represents the mean of three individual experiments. Plasmid transfection efficiency was 32.28±0.26% (p<0.05).

To investigate the functional roles of Wnt1 during apoptosis in CSCs, a Wnt1 gene expression vector was constructed and transfected into mCSCs. After transfection, Wnt1 expression levels were examined by real-time PCR and flow cytometry. Results showed that Wnt1 expression increased over time, levels at 72 h after transfection being 4.45-fold and 2.21-fold compared with the levels at 24 h and 48 h, respectively (Figure 2A). Because Wnt1 gene expression peaked at 72 h after transfection, the transfected CSCs were collected at 72 h to investigate transfection efficiencies by flow cytometry, which was 32.28±0.26% (Figure 2B).

### H_2_O_2_-induced Apoptosis in mCSCs

**Figure 3:**
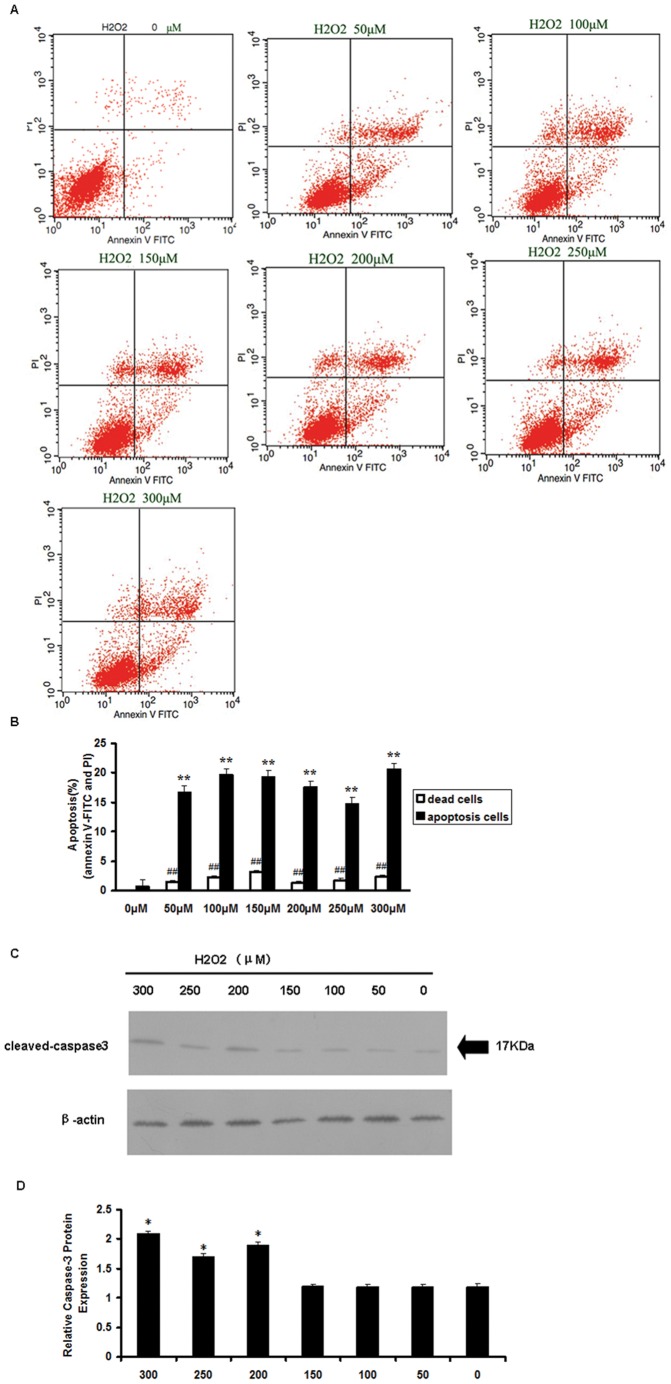
H_2_O_2_ induced apoptosis in mCSCs. (A) mCSCs were treated with a range of H_2_O_2_ concentrations (0, 50, 100, 150, 200, 250 and 300 µM) for 2 h. Cells were then stained with annexin V and PI and analyzed by flow cytometry. Apoptotic cells are localized in the lower right (early apoptosis) and upper right (late apoptosis) quadrants of the dot-plot graph using annexin V vs. PI. Dead cells are localized in the upper left quadrant. (B) Quantitative analysis of annexin V^+^ PI^−^ and annexinV^+^ PI^+^ CSCs by flow cytometry. (C) H_2_O_2_ induced caspase 3 activation in mCSCs. After H_2_O_2_ treatment using different concentration, cells were harvested and western blots were performed to compare the changes in caspase 3 activation. Western blot showed an increase in cleaved caspase 3 after treatment with a range of H_2_O_2_ concentration (from 0 µM to 300 µM), in a dose-dependent manner. (D) Densitometric analysis of cleaved caspase 3. (B and D) Bar graphs represent the mean values of triplicate measurements ± SD. *P<0.05; **P<0.01, compared with the control group (0 µM H_2_O_2_).

In order to study H_2_O_2_-induced apoptosis, we treated mCSCs with various concentrations of H_2_O_2_ for 2 h and examined cell apoptosis by flow cytometry. Cell apoptosis was measured by annexin V-FITC, which binds to phosphatidylserine residues that are redistributed from the inner to the outer leaflet of the cell membrane as an early event in apoptosis. After loss of membrane integrity, PI may also enter the cell and intercalate into DNA. [25] At concentrations ranging from 50 to 300 µM for 2 h, the proportions of annexin V-FITC-positive cells were 16.65±1.24%, 19.63±1.02, 19.21±1.22%, 17.55±1.13%, 14.74±1.09% and 18.54±1.13% respectively, compared to control (less than 0.7%). The proportions of annexin V^−^ PI^+^ (necrotic cells) were 1.37±0.32%, 2.26±0.18%, 3.14±0.21%, 1.27±0.24%, 1.64±0.47% and 2.38±0.17%, respectively, compared to control (less than 0.02%). We then used H_2_O_2_ at a 200 µM concentration to treat the mCSCs to induce apoptosis, since this concentration generated fewer dead cells. Results suggested that H_2_O_2_ treatment induced apoptosis in CSCs (Figure 3A).

To determine whether caspase 3 is involved in H_2_O_2_-induced apoptosis, we treated the cells with various concentration of H_2_O_2_ for 2 h. Then, the CSCs were collected to detect the caspase 3 activity by western blot. Results showed that caspase 3 activities were increased with increasing H_2_O_2_ concentrations, in a dose-dependent manner (Figure 3C). These results indicate that the H_2_O_2_-induced apoptosis may depend on caspase 3 activity.

### Overexpression of pEGFP-C3-Wnt1 Provides Additional Protection to mCSCs Against H_2_O_2_-induced Apoptosis

**Figure 4:**
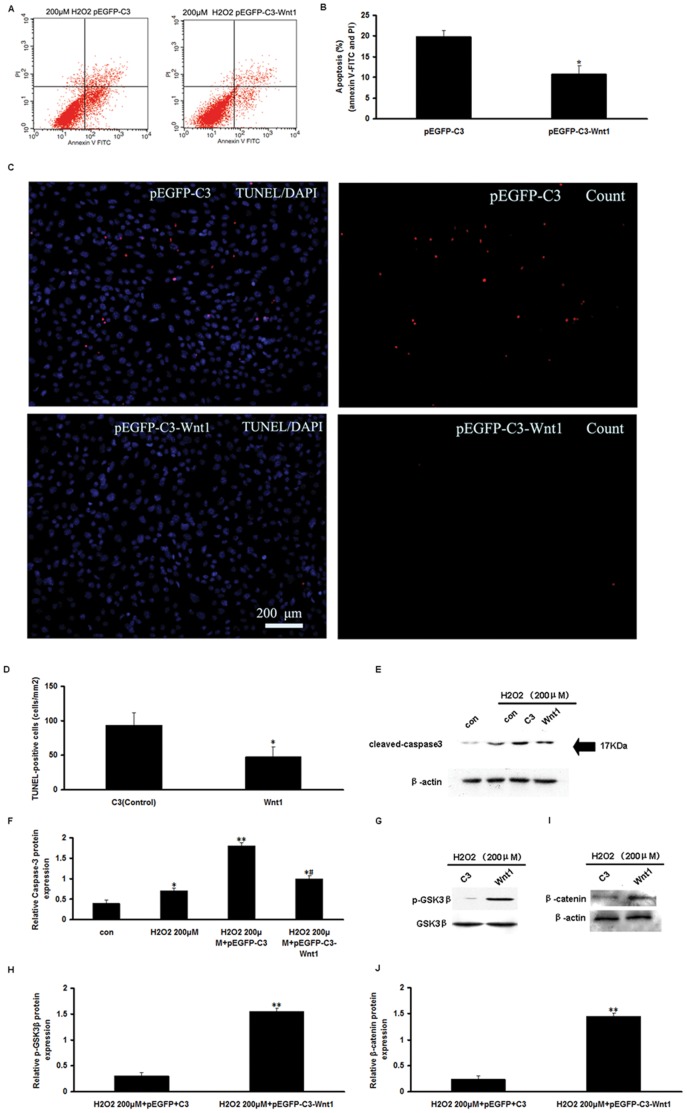
Overexpression of pEGFP-C3-Wnt1 prevented H_2_O_2_-induced apoptosis in mCSCs. (A) Representative annexin V/PI flow cytometry analysis of mCSCs. (B) Quantification of analysis of annexin V^+^/PI^−^ and annexin V^+^/PI^+^ mCSCs by flow cytometry. (C) Representative TUNEL-staining photographs for C3-mCSCs (control) and Wnt1-mCSCs. (D) Comparison of the numbers of TUNEL-positive cells from the two groups. *P<0.01 vs. the control group. (E) Representative immunoblot images of cleaved caspase 3 protein. Caspase 3 was activated after H_2_O_2_ treatment, but was decreased in Wnt1-CSCs. (F) Densitometric analysis of cleaved caspase 3 proteins from Figure 4E. (G) Representative immunoblot images of p-GSK3β and GSK3β proteins. (H) Densitometric analysis of p-GSK3β proteins from Figure 4G. (I) Representative immunoblot images of β-catenin and β-actin proteins. (J) Densitometric analysis of β-catenin protein from Figure 4I. (B, D, F, H and J) Bar graphs represent the mean values of triplicate measurements ± SD. *P<0.05 vs. the control group treated without 200 µM H_2_O_2_; # P<0.05 vs. the C3-CSCs group treated with 200 µM H_2_O_2_. **P<0.01 vs. the C3-CSCs group treated with 200 µM H_2_O_2_.

In order to determine the effects of Wnt1 gene expression on H_2_O_2_-induced mCSCs apoptosis, mCSCs were transfected with the Wnt1 plasmid (Wnt1-CSCs) 72 h before treatment with 200 µM of H_2_O_2_ for 2 h. As shown in Figures 4A and B, Wnt1 partly blocked H_2_O_2_-induced apoptosis (10.68±2.17%), compared to control C3-CSCs (19.77±1.6%). Apoptotic cells were also recognized by the identification of DNA fragmentation using the TUNEL assay. Wnt1-CSCs reduced TUNEL-positive cells compared to C3-CSCs (48±14 versus 94±18, p<0.05, Figures 4C and D). These results suggest that Wnt1 gene effectively prevented H_2_O_2_-induced CSCs apoptosis.

We next examined caspase 3 activation in H_2_O_2_-stimulated Wnt1- and C3-CSCs. Western blotting analysis revealed that the amounts of cleaved caspase 3 in H_2_O_2_-stimulated Wnt1-CSCs were decreased by 1.21-fold compared to C3-CSCs. These data imply that Wnt1 blocked the caspase 3-mediated apoptosis.

To investigate the molecular mechanism by which Wnt1 exerts its anti-apoptotic effects, the activation of the canonical Wnt pathway was examined. The canonical Wnt pathway involves the interaction of Wnt with the Frizzled receptor, resulting in the inhibition of GSK-3β by an unclear mechanism involving the phosphoprotein Dishevelled. We thus analyzed GSK3β activation by western blotting analysis with a phospho-GSK3β (p-GSK3β)-specific antibody. Results showed that Wnt1 significantly increased the amounts of p-GSK3β compared to the control cells (p<0.05, Figures 4G and H). These results indicate that Wnt1 prevented H_2_O_2_-induced CSCs apoptosis, at least in part, by altering caspase 3 activation.

β-catenin can be used as a signature for the activation of the canonical Wnt pathway. Therefore, we analyzed β-catenin expression by western blot analysis. As expected, Wnt1 increased the amounts of β-catenin compared with the control cells (p<0.05, Figures 4I and J). These data suggested that the effects of Wnt1 on mCSCs were regulated via the GSK3β/β-catenin pathway, mimicking canonical Wnt signaling.

### Inhibition of Wnt Pathway by SFRP2 in mCSCs Reduced Resistance to H_2_O_2_-mediated Apoptosis

**Figure 5:**
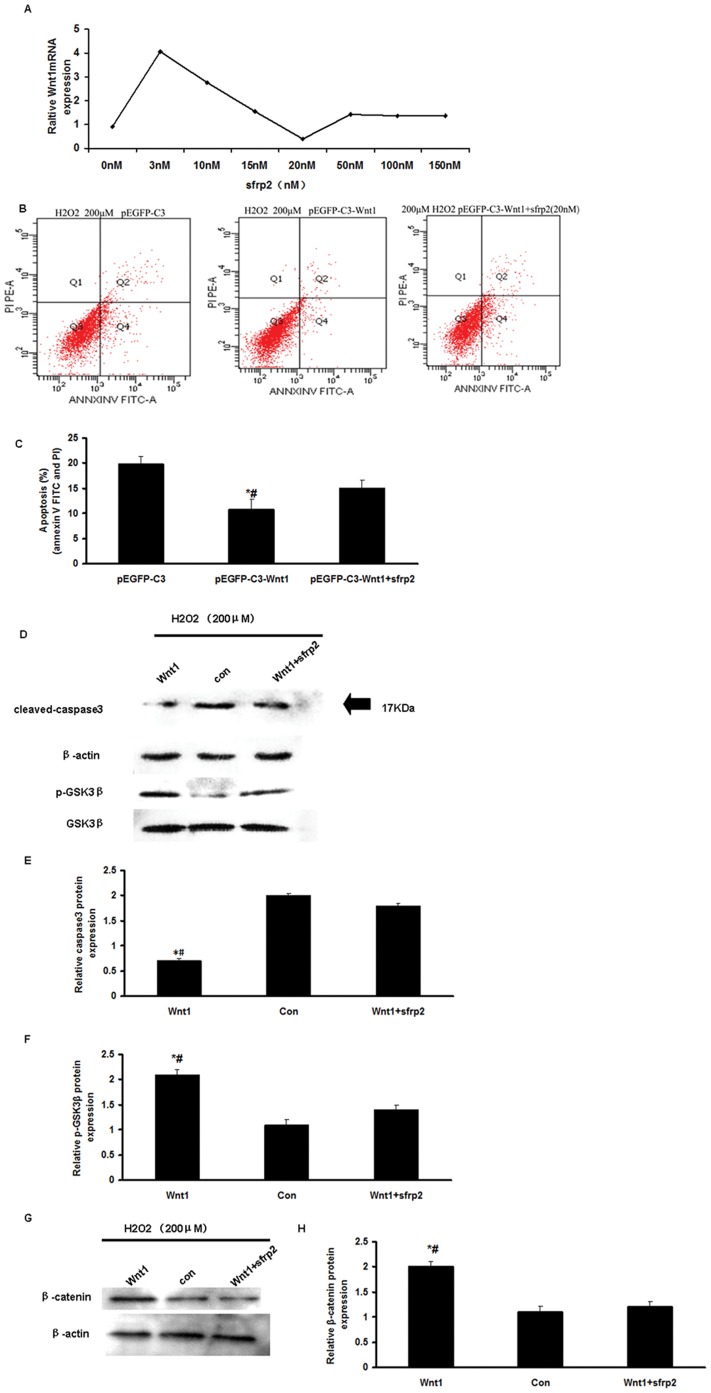
Inhibition of the Wnt pathway by SFRP2 reduced mCSCs’ resistance to H_2_O_2_-induced apoptosis. (A) Total mRNA was isolated from mCSCs treated with a range of concentrations of the SFRP2 protein (0, 3, 10, 15, 20, 50, 100 and 150 nM) for 24 h in serum-free medium. Real-time PCR was performed to quantify Wnt1 mRNA. (B) Representative annexin V/PI flow cytometry analysis of mCSCs. (C) Analysis of annexinV^+^/PI^−^ and annexin V^+^/PI^+^ mCSCs by flow cytometry. (D) Representative immunoblot images of cleaved caspase 3 and p-GSK3β proteins. Caspase 3 was activated after H_2_O_2_ treatment, but was decreased in C3-CSCs. (E) Densitometric analysis of cleaved caspase 3 protein from Figure 4D. (F) Densitometric analysis of p-GSK3β protein from Figure 4D. (G) Representative immunoblot images of β-catenin proteins. (H) Densitometric analysis of β-catenin protein from Figure 4G. (C, E, F and H) Bar graphs represent the mean values of triplicate measurements ± SD. *P<0.01 vs. the C3-CSCs group; #P<0.01, vs. SFRP2+Wnt1-CSCs.

To further elucidate the role of the Wnt pathway on the prevention of H_2_O_2_-induced mCSCs apoptosis, we examined mCSCs apoptosis and GSK3β activity in SFRP2-treated mCSCs. Wnt1-CSCs were first pretreated with various concentrations of SFRP2 (from 3 to 150 nM) for 24 h, followed by a treatment with H_2_O_2_ (200 µM for 2 h); Wnt1 mRNA expression levels were then quantified by real-time PCR (Figure 5A). It revealed that Wnt1 mRNA expression reached a minimum at 20 nM of SFRP2 and a maximum at 3 nM (Figure 5A). Results show that pretreatment with SFRP2 decreased Wnt1 mRNA expression in a dose-dependent manner. We then pretreated Wnt1-CSCs with 20 nM of SFRP2 for 24 h, followed by a treatment with H_2_O_2_ (200 µM for 2 h) and the apoptosis was quantified by flow cytometry. Results indicated that SFRP2 increased apoptosis, including both early (annexin V-FITC^+^ PI^−^ cells) and late apoptosis (annexin V-FITC^+^ PI^+^ cells). The proportion of annexin V-FITC^+^ SFRP2-treated cells was 14.99±1.7%, compared to untreated cells (10.68±2.17%).

We also detected caspase 3 protein levels in mCSCs with or without SFRP2 treatment. Results clearly showed that caspase 3 protein levels were obviously lower in the H_2_O_2_-induced apoptosis cells, compared to control cells. However, caspase 3 protein levels were not different in SFRP2-treated cells compared to control cells (p<0.05, Figure 5D and E). We thus analyzed GSK3β activation with p-GSK3β-specific antibody and β-catenin expression by western blot analysis Results showed that p-GSK3β and β-catenin expressions were was inhibited by SFRP2 treatment (p<0.05, Figures 5D, F, G and H). Taken together, these data suggested that the inhibition of the Wnt pathway by SFRP2 in mCSCs reduced resistance to H_2_O_2_-induced apoptosis, at least in part, by altering caspase 3 activation.

### Knockdown of β-catenin Increased Apoptosis in H_2_O_2_-induced mCSCs

To assess if the canonical Wnt signaling is directly linked to mCSCs apoptosis, β-catenin knockdown was performed using siRNA. Optimal time was 48 h after transfection (data not shown).

**Figure 6:**
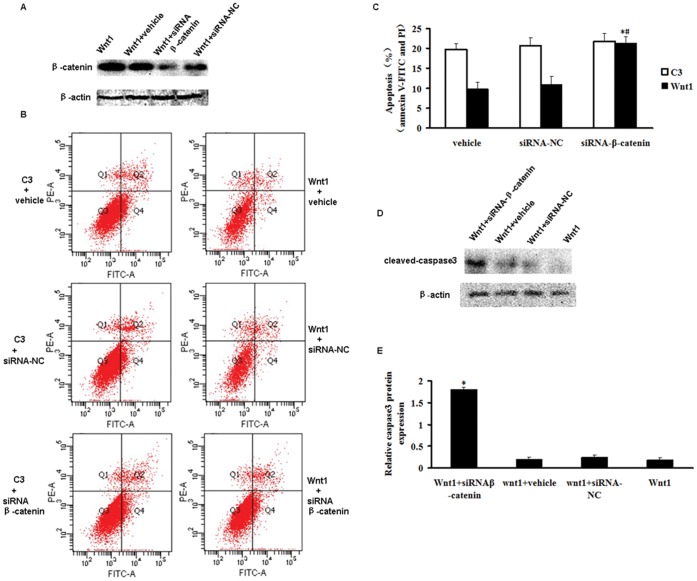
Inhibition of the Wnt pathway by β-catenin siRNA reduced mCSCs’ resistance to H_2_O_2_-induced apoptosis. CSCs were transfected with a siRNA against the β-catenin transcript. The siRNA-mediated transfection efficiency was demonstrated by fluorescence analysis (A). After 48 h, the transfected cells were harvested. Western blot analysis showed the suppression of β-catenin (B). (C) Representative annexin V/PI flow cytometry analysis of mCSCs. (D) Analysis of annexinV^+^/PI^−^ and annexin V^+^/PI^+^ mCSCs by flow cytometry. (D) Representative immunoblot images of cleaved caspase 3 protein. *P<0.05 vs. the vehicle+Wnt1-CSCs group; #P<0.05 vs. siRNA-NC+Wnt1-CSCs. (E) Densitometric analysis of cleaved caspase 3 protein from Figure 6D. *P<0.01 vs. the Wnt1-CSCs group. (D and F) Bar graphs represent the mean values of triplicate measurements ± SD.

Western blotting revealed that siRNA transfection decreased β-catenin protein level by 23-fold compared to siRNA-NC-treated cells (Figure 6A). Thus, silencing β-catenin with siRNA successfully reduced β-catenin protein expression.

We then pretreated Wnt1-CSCs with siRNA-β-catenin for 48 h, followed by a treatment with H_2_O_2_ (200 µM for 2 h); apoptosis was then quantified by flow cytometry. Results revealed that knocking-down β-catenin increased apoptosis, including both early (annexin V-FITC^+^ PI^−^ cells) and late apoptosis (annexin V-FITC^+^ PI^+^ cells). The proportion of annexin V-FITC^+^ siRNA-β-catenin-treated Wnt1-CSCs was 20.99±1.47%, compared with siRNA-NC-treated Wnt1-CSCs (11.68±2.17%) and vehicle-treated Wnt1-CSCs (10.45±1.76%). However, the proportion of annexin V-FITC^+^ was not different in siRNA-β-catenin-treated Wnt1-CSCs (20.99±1.47%) compared with siRNA-β-catenin-treated C3-CSCs (21.04±1.41%). Also, the proportion of annexin V-FITC^+^ was not different in siRNA-NC-treated Wnt1-CSCs (11.68±2.17%) compared with vehicle-treated Wnt1-CSCs (10.45±1.76%).

We also evaluated caspase 3 protein levels in Wnt-mCSCs treated with siRNA-β-catenin, vehicle alone and siRNA-NC, after a treatment with H_2_O_2_ (200 µM for 2 h). Results clearly showed that caspase 3 protein levels were higher in the siRNA-β-catenin group, compared with the siRNA-NC group. Caspase 3 protein levels were not different in the vehicle group compared with the siRNA-NC group (Figures 6D and E). Taken together, these data suggested that the inhibition of Wnt pathway by knocking-down the β-catenin protein in mCSCs reduced resistance to H_2_O_2_-induced apoptosis, at least in part, by altering caspase 3 activation.

## Discussion

In ischemic hearts, the native and exogenous stem cells are exposed to a number of pro-apoptotic or cytotoxic factors impairing cellular cardiomyoplasty. In this present study, we showed that the Wnt1 gene induces some defenses against apoptosis in mCSCs. The main findings from the present study are: 1) H_2_O_2_ treatment induces mCSCs apoptosis via the caspase 3 pathway, in a dose-dependent manner; 2) Wnt1 over-expression provides additional protection to mCSCs against H_2_O_2_-induced apoptosis; and 3) inhibition of the canonical Wnt pathway by SFRP2 or by knicking-down of β-catenin in mCSCs reduced cells’ resistance to H_2_O_2_-induced apoptosis. Overall, results showed that Wnt1 plays an important role in cardiac stem cells’ defenses against H_2_O_2_-induced apoptosis through the canonical Wnt signaling Wnt1/GSK3β/β-catenin.

Because of the role of bone marrow-derived Lin-/c-kit+ cells in myocardial regeneration [26], of the mesodermal origin of both the heart and the bone marrow, and of the use of c-kit as a hematopoietic stem cell marker [27], [28], [29], we decided to concentrate our efforts on the cardiac cells expressing this marker, i.e. the receptor for stem cell factor. We first showed that cardiac c-kit+ cells could be isolated and purified from early postnatal hearts using enzymatic dissociation and magnetic-activated cell sorting (MACS), producing a pure population of cells that can be used for further studies. The isolated c-kit+ cells population contained cells at several early stages of cardiac myogenic differentiation, as demonstrated by the expression of the transcription factors GATA4, and by the amounts of desmin protein in the cytoplasm. It should be noted that most, if not all, of the purified c-kit+ cells were negative for the endothelial/hematopoietic progenitor marker CD34. This phenotype strongly suggests that these cells are amplified myogenic precursors and/or progenitors derived from the activation of more primitive stem cells. The expression of transcription factors associated with early cardiac development, such as GATA4 [30] and the expression of muscle-specific desmin proteins by most of the cells (see Figure 1), are strong evidences supporting their cardiac myogenic potential and, most likely, their cardiac myogenic fate. With a few exceptions, freshly isolated c-kit+ cells were negative for mature myocyte markers such as heavy chain cardiac myosin and Tn-I. These results further showed that the c-kit+ cell population is heterogeneous in terms of differentiation stage and that some of them are already committed to the cardiac myogenic lineage. Most of c-kit+ cells expressed mesenchymal specific surface proteins, such as CD90 and CD29, suggesting that this pure population of cells was from mesodermal origin.

Native resident CSCs are a responsive stem cell reservoir within the adult myocardium. They may, therefore, offer distinct advantages over other adult stem cell types for cardiovascular therapy, being autologous, tissue-specific and pre-committed to the cardiovascular lineage. [31] However, despite their apparent therapeutic potential, some caution is warranted regarding c-kit^+^ CSCs, since they are very rare within the myocardium. However, the molecular cues and signaling pathways that regulating CSCs homeostasis in tissue injury, especially in MI, remain unclear.

Apoptosis of c-kit^+^ CSCs play a pivotal role in the pathogenesis of heart diseases. CSCs protection from H_2_O_2_-induced apoptosis may provide beneficial therapeutic intervention to successfully fight cardiovascular diseases, especially MI. In this study, we demonstrated that Wnt1 was able of saving c-kit^+^ CSCs from apoptosis induced by oxidative stress (H_2_O_2_), suggesting that Wnt1 may have a therapeutic use in the prevention and the treatment of cardiovascular diseases.

Very little is known about the apoptotic effects of H_2_O_2_ in c-kit^+^ CSCs. Our results indicate that H_2_O_2_ induced c-kit^+^ CSCs apoptosis by caspases-3 activation. Caspases belong to a family of specific cysteine proteases and are critical apoptosis mediators. Fourteen members of the caspase family have been identified so far. [32] Among them, caspase 3 is a primary apoptosis activator induced by a variety of stimuli, including H_2_O_2_. [33], [34] We showed that Wnt1 attenuated the activation of caspase 3 in a dose-dependent manner. The mechanisms by which H_2_O_2_ induces caspase activation in CSCs are not fully understood. This activation could be due to direct oxidative stress, or it could be mediated by mitochondria or by other mechanisms; any of these mechanisms might be inhibited by Wnt1. H_2_O_2_ exposure might be involved in cell signaling pathways that are crucial for determining whether a cell survives or dies.

A growing body of evidences revealed that Wnt might play a role in apoptosis and in the pathogenesis of a number of diseases. However, the existing evidences are conflicting. Ming et al. [35] reported that the activated Wnt pathway dysregulates the survival of hematopoietic progenitor cells by inducing the mitochondrial apoptotic pathway. On the other hand, Farhana et al. [36] showed that the inhibition of the Wnt/β-catenin pathway induced apoptosis and inhibited cell growth in pancreatic cancer cells. Similarly, we showed that the activation of Wnt pathway by Wnt1 increased cell survival. SFRPs are expressed in many cell types during embryogenesis [37] and participate in modulating Wnt-frizzled signaling [38] and apoptosis. [39] Also, SFRP2 is a modulator of the Wnt signaling pathway and is involved in development, apoptosis regulation and cancer progression. [39] In this present study, pEGFP-C3-Wnt1-transfected CSCs were exposed to different SFRP2 concentrations to H_2_O_2_. Our results showed that the inhibition of GSK3β activity by the Wnt antagonist SFRP2 reduced the resistance of cells to H_2_O_2_-induced apoptosis. Similar results were also obtained in β-catenin knockdown (siβ-catenin) experiments, in which the canonical Wnt signaling pathway was completely blocked. Taken together, these results suggest that Wnt1 is an apoptosis signal mediator in H_2_O_2_-stimulated CSCs.

In summary, the present study revealed a critical role for Wnt1 as a cardiac protective agent in the protection against H_2_O_2_-induced cell apoptosis.
